# Transient receptor potential vanilloid type 1 is expressed in the horizontal pathway of the vervet monkey retina

**DOI:** 10.1038/s41598-020-68937-9

**Published:** 2020-07-21

**Authors:** Joseph Bouskila, Catarina Micaelo-Fernandes, Roberta M. Palmour, Jean-François Bouchard, Maurice Ptito

**Affiliations:** 1 Departments of Psychiatry and Human Genetics, McGill University, Montreal, QC Canada; 2 Behavioral Science Foundations, St. Kitts, Saint Kitts and Nevis; 3 School of Optometry, University of Montreal, Montreal, QC Canada; 4 Department of Nuclear Medicine, University of Southern Denmark, Odense, Denmark

**Keywords:** Retina, Retina

## Abstract

The ubiquitous distribution of the classic endocannabinoid system (cannabinoid receptors CB1 and CB2) has been demonstrated within the monkey nervous system, including the retina. Transient receptor potential vanilloid type 1 (TRPV1) is a cannabinoid-like non-selective cation channel receptor that is present in the retina and binds to endovannilloids and endocannabinoids, like anandamide, 2-arachidonoylglycerol and N-arachidonoyl dopamine. Retinal expression patterns of TRPV1 are available for rodents and data in higher mammals like humans and monkeys are scarce. We therefore thoroughly examined the expression and localization of TRPV1 in the retina, at various eccentricities, of the vervet (*Chlorocebus sabeus*) monkey, using Western blots and immunohistochemistry. Our results demonstrate that TRPV1 is found mainly in the outer and inner plexiform layers, and in the retinal ganglion cell (RGC) layer with a higher density in the periphery. Co-immunolabeling of TRPV1 with parvalbumin, a primate horizontal cell marker, revealed a clear overlap of expression throughout the entire cell structure with most prominent staining in the cell body membrane and synaptic terminals. Furthermore, double labeling of TRPV1 and syntaxin was found throughout amacrine cells in the inner plexiform layer. Finally, double staining of TRPV1 and Brn3a allowed us to confirm its previously reported expression in the cell bodies and dendrites of RGCs. The presence of TRPV1 in the horizontal pathway suggests a function of this receptor in lateral inhibition between photoreceptors through the horizontal cells, and between bipolar cells through amacrine cells.

## Introduction

The transient receptor potential vanilloid type 1 (TRPV1) is a non-selective cation channel receptor that is expressed in numerous cell types throughout the entire body. TRPV1 is found in microglial cells in the brain and in the retina of several animal species^1–3^. In the retina, TRPV1 has been studied mostly in rats, rabbits and fishes, but in primates and humans, data are scarce. For example, in the goldfish retina, TRPV1 is expressed in three anatomically distinct amacrine cell types^4^ and, in rats, it is localized in the ganglion cell layer (GCL), the optic nerve head, and in the optic nerve itself^5^. Moreover, in the same species, TRPV1 is located in small bundles of RGC axons in the peripheral retina and is densely expressed in large axon bundles close to the optic nerve^6^. TRPV1 was localized in rabbit Müller cells^7^ but not in rats^5^. In genetically manipulated TRPV1Cre:Ai9 and TRPV1Cre:Ai3 mice^8,9^, TRPV1 was found in RGCs but not amacrine cells^10^. As for the primate retina, only one study has located TRPV1 receptor in the GCL, but the exact retinal locus was not reported, neither the complete specific retinal cell types expressing it. This receptor was also found in the RGC dendrites, with diffuse labeling in the soma. In the human retina, TRPV1 was slightly more diffuse in the RGC cytoplasm compared to the macaque^11^. Differences in the expression of TRPV1 are probably due to the lack of specificity of the antibodies^12,13^ and the interspecies’ differences.

In TRPV1 knockout mice (*trpv1*^−/−^) and with pharmacological blockade of TRPV1 in normal mice, an accelerated degeneration of RGCs occurs in a model of high intraocular pressure (IOP)^14^. Moreover, in *trpv1*^−/−^ RGCs lacked compensatory transient increase in spontaneous action potentials and necessitated higher depolarization to reach firing threshold when exposed to high IOP^11,15^. It was also reported that in microglia, TRPV1 was involved in the modulation and regulation of Ca^2+^ activity. Indeed, TRPV1 activation was linked with a strong conductance of Ca^2+^ that is related to the apoptosis of neurons and glial cells^2,6,16–18^. Furthermore, endogenous cannabinoids like anandamide (AEA) and 2-arachidonoylglycerol (2-AG), ligands of TRPV1, might play a protective role against ischemic injury and excitotoxicity^11,15,19,20^. TRPV1 has also several modulatory functions through multimodal integration of chemical stimuli such as capsaicin and endocannabinoids^21–25^.

From all the reported studies, there is not a precise localization of TRPV1 in the various components of the retinal mosaic and there are also contradictory results that might be attributed to interspecies’ differences and specificity of the antibody used. Moreover, studies on monkeys are scarce and the complete profile of cells expressing TRPV1 is lacking. In this study, we have thoroughly mapped TRPV1 expression in all cell types of the entire retina (from the center to the periphery) of the vervet monkey using Western blots and immunohistochemistry. Our results demonstrated that TRPV1 was mainly expressed in the horizontal system of the retina (horizontal and amacrine cells) that adds to the previously reported data by our laboratory on the endocannabinoid receptor expression in the vertical retinal system (from photoreceptors to ganglion cells). We also propose a model regarding the putative role of TRPV1 in a two-stage lateral inhibition, one at the receptor level through horizontal cells and the other at the bipolar cells’ level through amacrine cells, that might contribute to perceptual contrast enhancement.

## Materials and methods

### Tissue preparation

The eyes were taken from 5 adult vervet monkeys (*Chlorocebus sabeus*; aged 3–4 years) and were part of ongoing research projects approved by the University of Montreal and the Behavioural Science Foundation Animal Care Committee. All methods were performed in accordance with the ARRIVE guidelines and the regulations of the Canadian Council on Animal Care. No animals were specially sacrificed for this study. The description of the in vivo animal preparation has been described in previous publications^26–29^, and will be described briefly here. The animals were deeply anaesthetized with a lethal dose of sodium pentobarbital (25 mg/kg, intravenous) and perfused through the heart with a solution of phosphate buffered saline (PBS; pH 7.4), followed by 4% paraformaldehyde (PFA) solution in PBS. The eyes were then removed and the anterior segment of the eye, namely the cornea, iris, ciliary body, and lens, as well as the vitreous humour were cut away. The eyecups were bathed in 4% PFA (prepared in 0.1 M PBS, pH 7.4) and left in the solution. The retina was dissected free from the eyecup in a PBS medium. It was laid flat, and by brushing gently with filter paper, the vitreous body was removed. Samples of the retina were taken at the center, middle, and periphery. Each sample was then cryoprotected in 30% sucrose overnight and embedded in Shandon embedding media at − 65 °C. The blocks were cut in 20 μm sections at -18 °C with a Leica CM3050S cryostat and mounted onto gelatinized subbed glass slides. After air drying, the slides were stored at − 80 °C until further processing.

### Immunofluorescence

Single- and double-labeling of the retina were performed according to previously published methods^26–28^. Briefly, the sections were rinsed 3 × 5 min in 0.1 M Tris buffer, pH 7.4/0.03% Triton and blocked for 90 min in a blocking solution (10% normal donkey serum (NDS), 0.1 M Tris buffer/0.5% Triton). Sections were then incubated with primary antibodies prepared in blocking solution overnight at room temperature. The TRPV1 antibody (#NB100-1617, Novus Biologicals, Littleton, CO, USA) was also used conjointly with one of these known specific retinal cell type markers, namely protein kinase C (PKC), syntaxin, parvalbumin (PV), and Brain-specific homeobox/POU domain protein 3A (Brn3a) (see Table 1). The next day, sections were washed for 10 min and 2 × 5 min in 0.1 M Tris/0.03% Triton. They were then blocked in 10% NDS, 0.1 M Tris /0.5% Triton for 60 min and incubated with secondary antibodies for one hour (Alexa 488 donkey anti-mouse or Alexa 647 donkey anti-mouse and Alexa 647 donkey anti-rabbit or Alexa 488 donkey anti-rabbit at 1:200, all prepared in blocking solution). Sections were counterstained with Sytox Orange Nucleic Acid Stain (1:10,000; Molecular Probes, Inc., Eugene, OR, USA) to properly align retinal sections for confocal analysis, and washed again in Tris buffer, and coverslipped with Fluoromount-G™ Mounting Medium (SouthernBiotech, Birmingham, AL, USA).

**Table 1:** List of antibodies.

Antibody*	Immunogen	Source^†^	Working dilution	MW (kDa)
Brn3a	Amino acids 186–224 of Brn3a protein	Mouse monoclonal, EMD Millipore Corporation, MAB1585, AB_94166	I: 1:50	46
GS	Full protein purified from sheep brain	Mouse monoclonal, Clone GS-6, EMD Millipore Corporation, MAB302, AB_2110656	I: 1:200	45
PKCα	Epitope mapping between amino acids 645–672 at the C-terminus of PKCα of human origin	Mouse monoclonal, Clone H-7, Santa Cruz Biotechnology, sc-8393, AB_628142	I: 1:200	80
PV	Parvalbumin purified from carp muscles	Mouse monoclonal, Swant, 235, AB_10000343	I: 1:200	12
Syntaxin	Synaptosomal plasma fraction of rat hippocampus^**‡**^	Mouse monoclonal, Clone HPC-1, Sigma-Aldrich, S0664, AB_477483	I: 1:200	35
TRPV1	Amino acids 4–21 of rat TRPV1	Rabbit polyclonal, Novus, NB100-1617, AB_10002124	I: 1:200 W: 1:500	100
Alexa Fluor 488 donkey anti-mouse	Mouse Gamma Immunoglobins Heavy and Light chains	Donkey polyclonal, Invitrogen, A-21202, AB_141607	I: 1:200	N/A
Alexa Fluor 488 donkey anti-rabbit	Rabbit Gamma Immunoglobins Heavy and Light chains	Donkey polyclonal, Invitrogen, A-21206, AB_2535792	I: 1:200	N/A
Alexa Fluor 647 donkey anti-mouse	Mouse Gamma Immunoglobins Heavy and Light chains	Donkey polyclonal, Invitrogen, A-31571, AB_162542	I: 1:200	N/A
Alexa Fluor 647 donkey anti-rabbit	Rabbit Gamma Immunoglobins Heavy and Light chains	Donkey polyclonal, Invitrogen, A-31573, AB_2536183	I: 1:200	N/A

*GS glutamine synthetase, *I* immunohistochemistry, *MW* molecular weight, *PKCα* protein kinase C, *PV* parvalbumin, *TRPV1*, transient receptor potential vanilloid 1, *W* Western blot.

^†^The source column indicates the host species, commercial company, catalog reference and RRID. The clone designation is given for monoclonal antibodies when available.

^‡^See^39^.

### Western blotting

Western blot analysis was performed according to previously published methods^28^. Briefly, frozen retinal tissue was homogenized by hand using a sterile pestle in a radioimmunoprecipitation assay buffer (150 mM NaCl, 20 mM Tris, pH 8.0, 1% NP-40, 0.1% sodium dodecyl sulfate (SDS), 1 mM EDTA), supplemented with protease inhibitors (Pierce Protease Inhibitor Tablets #88266, Thermo Fisher Scientific, Rockford, IL, USA) and phenylmethylsulfonyl fluoride (0.2 mg/ml; Roche Applied Science, Laval, QC, Canada). At 4 °C for 10 min, the samples were then centrifuged, and the supernatant was extracted and stored at − 20 °C until further processing. Protein content was equalized using a Thermo Scientific Pierce BCA Protein Assay Kit (Fischer Scientific, Ottawa, ON, Canada). Ten micrograms of protein homogenate per sample were loaded for electrophoresis in a 10% sodium dodecyl sulfate (SDS)-polyacrylamide gel. Proteins were then transferred to a polyvinylidene difluoride (PVDF) membrane using the Trans-Blot Turbo Transfer System (Bio-Rad, Hercules, CA, USA). Upon recovering and washing the membrane in TBST (5 M NaCl, 1 M pH 8 Tris, 50% Tween-20) five times for five minutes each time, the membranes were blocked for one hour in 5% skim milk in TBST. Thereafter, the membrane was left incubating overnight with the rabbit anti-TRPV1 primary antibody in blocking solution in a 1:500 dilution at 4ºC. On the following day, the membrane was washed five times for five minutes in TBST before and after a 2-h incubation with horseradish peroxidase (HRP) conjugated donkey anti-rabbit secondary antibody in blocking solution in a 1:5,000 dilution at room temperature. Enhanced chemiluminescence (ECL) Clarity Western blot substrate was used for protein detection (Bio-Rad, Hercules, CA, USA). Proteins were visualized using the ChemiDoc Imaging Software (Bio-Rad, Hercules, CA, USA). For the control condition, the same protocol was ran simultaneously as described, excepting that the anti-TRPV1 primary antibody was preincubated with its blocking peptide (BP; #NB100-1617PEP, Novus Biologicals, Littleton, CO, USA) in a 1:10 dilution for 1 h.

### Confocal microscopy

Immunofluorescence images were taken according to published methods^28,29^. Using a Leica TCS SP2 confocal laser-scanning microscope (Leica Microsystems, Exton, PA) or an Olympus FV3000 confocal laser-scanning microscope (Olympus Canada, Richmond Hill, ON, USA), images were obtained sequentially from the green, blue or far-red channels on optical slices of less than 0.9 μm of thickness. All photomicrograph adjustments, including size, color, brightness, and contrast were done with Adobe Photoshop (CC, Adobe Systems, San Jose, CA) equally for all images for each condition, and then exported to Adobe InDesign (CC, Adobe Systems, San Jose, CA), where the final figure layout was completed.

### Optical density measurements

Confocal micrographs were first converted to an 8-bit grayscale image setting to visualize TRPV1 immunolabeling throughout the retinal layers. Using the public domain software Fiji (ImageJ, version 2.0.0, NIH Image, Bethesda, Maryland), mean gray values for all images to be quantified were measured. The gray spectrum values were generated from the pixel intensity (arbitrary value 0–255, 0 representing black and no signal, and 255 representing white and very strong signal).

### Statistical analysis

A one-way analysis of variance (ANOVA) was conducted to evaluate the differences in the mean relative optical densities of TRPV1 through the six different layers of the retina (N = 4 measurements). For each layer, a one-way ANOVA was also performed to compare TRPV1 labeling intensity through three distinct retinal eccentricities. Scheffe’s test was used for all post-hoc comparisons.

## Results

### TRPV1 antibody specificity

**Figure 1:**
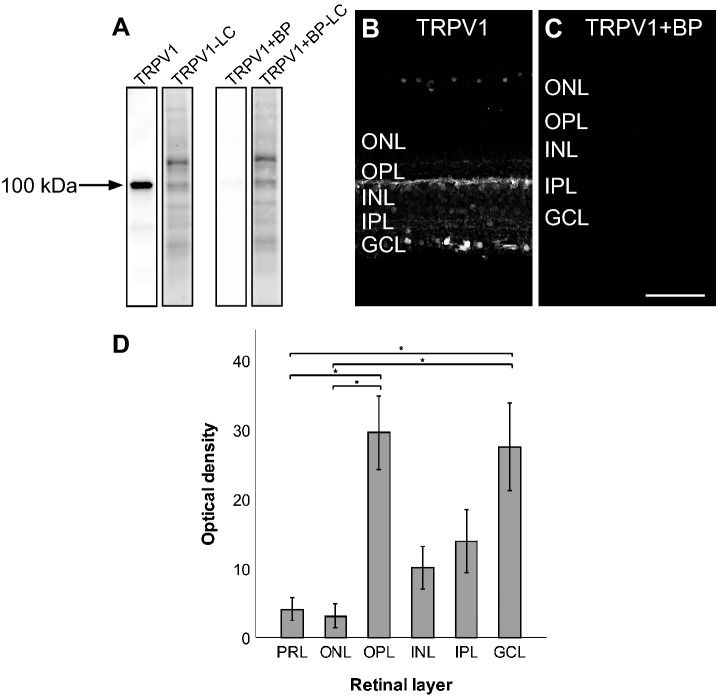
Characterization of the TRPV1 antibody in the monkey retina. Western blot analysis of total protein samples from a vervet monkey retina, showing detection of the expected protein band at 100 kDa (**A**). The band was not detected when the antibody was pre-incubated with its corresponding TRPV1 blocking peptide (BP) at the ratio 1:10 (**A**). All lanes contained 10 μg of total protein. Immunohistochemistry on vervet retinal tissue with the antibody raised against TRPV1 revealed a unique staining profile (**B**). When the TRPV1 antibody was pre-incubated with its corresponding BP, it revealed an absence of staining in the vervet retinas (**C**). Quantitative analysis of the TRPV1 fluorescent signal through the six different retinal layers reveals a relatively higher signal intensity in the OPL and the GCL layers compared to the photoreceptor (PRL) and the outer nuclear (ONL) layers (P < 0.05) (**D**). *TRPV1* transient receptor potential vanilloid type 1, *BP* blocking peptide, *LC* loading control, *PRL* photoreceptor layer, *ONL* outer nuclear layer, *OPL* outer plexiform layer, *INL* inner nuclear layer, *IPL* inner plexiform layer, *GCL* ganglion cell layer. Scale bar = 75 μm. Mean optical density ratios ± standard error of the mean (SEM) are depicted.

Western blot analysis demonstrates binding specificity of the TRPV1 antibody. A protein band is detected around 100 kDa, in accordance with the previously reported molecular weight for TRPV1^14^. The signal is abolished when samples were incubated with its blocking peptide (Fig. 1A). We also tested for antibody specificity using immunohistochemistry. Retinal sections immunolabeled for TRPV1 showed expression in the outer plexiform (OPL), the inner plexiform (IPL), and ganglion cell layers (GCL) (Fig. 1B). Pre-incubation with the corresponding TRPV1 blocking peptide completely abolished antibody signal (Fig. 1C). The OPL and the GCL exhibited higher labeling intensity as compared to the photoreceptor (PRL) and the outer nuclear (ONL) layers (P < 0.05) (Fig. 1D).

### TRPV1 immunoreactivity throughout the vervet retina

**Figure 2:**
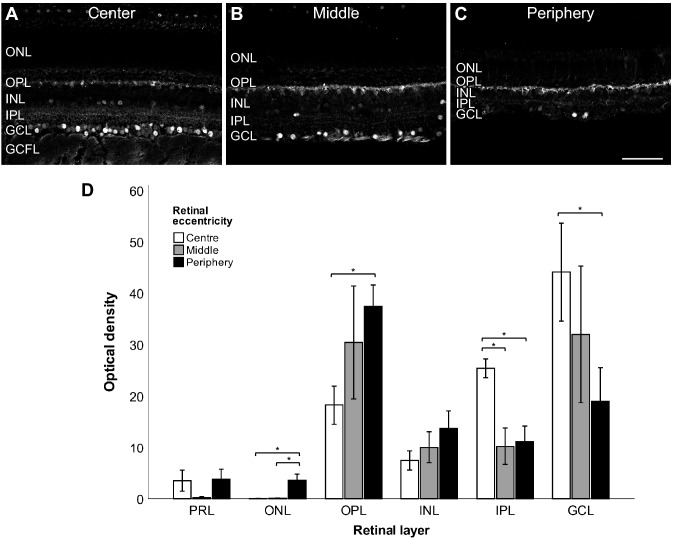
Labeling pattern of TRPV1-IR throughout the vervet retina. Confocal micrographs taken from the center (**A**), middle (**B**), and peripheral retina (**C**). Quantitative analysis of the TRPV1 fluorescent signal through the three distinct retinal eccentricities shows relatively higher central signal intensity in the IPL and the RGC layers, and greater peripherical labeling in the OPL and the ONL layers (P < 0.05) (**D**). *PRL* photoreceptor layer, *ONL* outer nuclear layer, *OPL* outer plexiform layer, *INL* inner nuclear layer, *IPL* inner plexiform layer, *GCL* ganglion cell layer, *GCFL* ganglion cell fiber layer. Scale bar = 75 µm. Mean optical density ratios ± standard error of the mean (SEM) are depicted.

TRPV1-immunoreactivity (IR) was present throughout the retina: center (Fig. 2A), middle (Fig. 2B), and periphery (Fig. 2C). A strong TRPV1 signal was localized in the lateral pathway, particularly in the IPL and OPL. Layer-specific differences were found on the relative distribution of TRPV1 through the three distinct retinal eccentricities, with the IPL and the GCL layers displaying higher signal intensity in the central retina, and the OPL and the ONL layers showing stronger peripherical TRPV1 labeling (Fig. 2D).

### Double-labeling immunohistochemistry

In order to verify the retinal cell type expression, double immunostaining was carried out for TRPV1 and a molecular marker for specific primate retinal cells. A consistent staining pattern across all five monkey retinas was found for each double staining. Although labeling was observed in all retinal layers, TRPV1-IR was most prominent in the horizontal pathway, namely horizontal and amacrine cells.

### Distribution of TRPV1 in the various cells of the retina

#### TRPV1 is found in retinal ganglion cells

**Figure 3:**
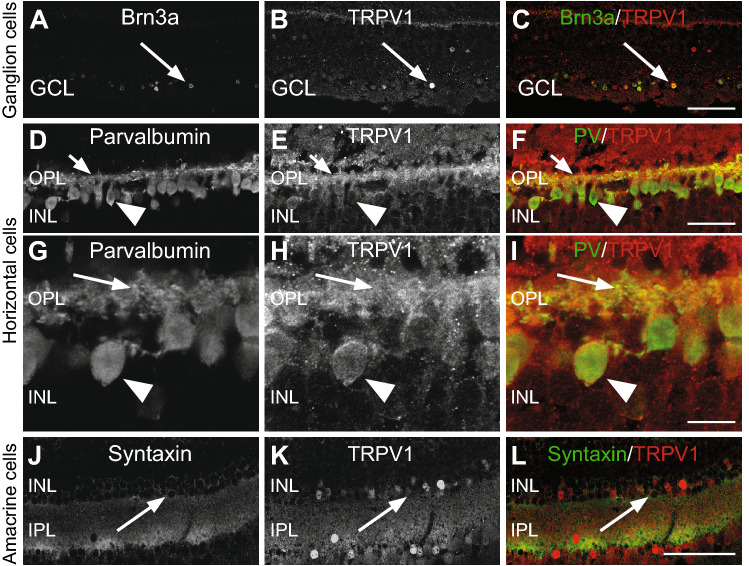
Double-label immunofluorescence illustrating immunolabeling of TRPV1 in ganglion cells, horizontal cells, and amacrine cells. (**A-C**) The antibody against Brn3a labels the nucleus of ganglion cells in the monkey retina, and these cells were strongly TRPV1 immunoreactive. Arrows indicate Brn3a-positive ganglion cells that are TRPV1 immunoreactive. Each protein expression is presented alone in grayscale: Brn3a (**A**) and TRPV1 (**B**); then, the two are presented merged (**C**), Brn3a in green and TRPV1 in red). (**D-I**) TRPV1 is expressed in horizontal cell synaptic terminals (arrows in OPL) and cell bodies (arrowheads in the INL). Each expression is presented alone in grayscale: PV (**D,G**), TRPV1 (**E,H**); then, the two are presented merged (**F,J**). (**J**–**L**) Arrows show the expression of TRPV1 in the amacrine cell bodies (INL). Each expression is presented alone in grayscale: syntaxin-positive amacrine cells (**J**) and TRPV1 (**K**); then, the two are presented merged (**L**). *INL* inner nuclear layer, *IPL* inner plexiform layer, *OPL* outer plexiform layer, *GCL* ganglion cell layer. Scale = 75 μm for (**A**–**C**) and (**J**–**L**), 30 μm for (**D**–**F**) and 10 μm for (**G**–**I**).

The widespread TRPV1-positive cell bodies in the ganglion cell layer suggests specific ganglion cell labeling (Fig. 3A–C). We verified this result with double-labeling of TRPV1-IR with Brn3a-IR, that characterizes ganglion cells in the retina of mammals, including our species (vervet monkey)^26,28^. Most cell bodies Brn3a-positive were also double-labeled for TRPV1 immunoreactivity. This included all the ganglion cell fibers. Most prominent staining appears to be in the central retina.

#### Horizontal cells express TRPV1

Since TRPV1-IR is co-localized with PV-immunoreactive cells, it indicates that horizontal cells are indeed TRPV1-positive. The PV-immunoreactivity is generally linked to similar staining of 2 specific classes of horizontal cells in the monkey retina (arrows), namely H1 and H2, as reported by Röhrenbeck et al. (1987). Obvious co-localization was observed in both horizontal cell types in the retina of vervet monkeys^30^ (Fig. 3D–I).

#### TRPV1 is localized in amacrine cells

To evaluate TRPV1-IR expression in amacrine cells, we used the monoclonal antibody HPC-1 that recognizes syntaxin in these cells. All amacrine cells showed expression of TRPV1-IR (Fig. 3J–L) even though variations in the intensity of immunolabeling were noticed.

#### TRPV1 is absent in rod bipolar cells

**Figure 4:**
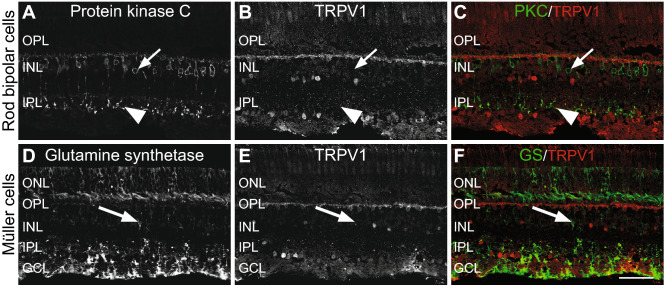
Double-label immunofluorescence showing no signal for TRPV1 in rod bipolar cells and Müller cells. TRPV1 is neither expressed in the cell bodies (small arrows in the upper INL) of bipolar cells nor their synaptic terminals at the edge of the OPL (arrowheads). Each protein expression is presented alone in grayscale: PKC (**A**) and TRPV1 (**B**); then, the two are presented merged (**C**), PKC in green and TRPV1 in red. Long arrows show specifically that the Müller cell bodies do not express TRPV1. Each protein expression is presented alone in grayscale: glutamine synthetase (**D**) and TRPV1 (**E**); then, the two are presented merged (**F**), glutamine synthetase in green and TRPV1 in red. Scale = 75 μm for (**A**–**F**).

Cell fibers in the proximal INL that were TRPV1-immunoreactive looked like PKC-immunoreactive rod bipolar cell axons in the central retina. However, no PKC-IR rod bipolar cells, including their cell bodies, axons, and axon terminals, were co-localized with TRPV1 labeling, confirming that TRPV1 was absent in rod bipolar cells (Fig. 4A–C).

#### TRPV1 is not expressed in the retinal glia (Müller cells)

In order to assess the expression of TRPV1 in Müller cells, we used double labeling of TRPV1 and glutamine synthetase (GS), a specific marker of the soma and processes of Müller cells^31–33^. Müller cells cover the entire retina and their extensions spread out into the GCL to form characteristic end-feet. GS-positive cell bodies and processes of Müller cells did not show TRPV1 immunoreactivity (Fig. 4D–F).

## Discussion

Our anatomical results indicate the existence of TRPV1 in the horizontal pathway of the vervet monkey retina, extending further the results obtained by others^14,34^. Moreover, TRPV1 spans the whole vervet monkey retina, from its center to the periphery. While TRPV1 has been localized in a few retinal cells among different species^14,34^, this study is the first to show its precise expression in other retinal layers besides the GCL. Moreover, our results clarify the expression and localization of TRPV1 in the retina, mostly in the inhibitory cells of the lateral pathway. This localization extends further our understanding of the distribution of the endocannabinoid receptors in the monkey retina. The presence of TRPV1 in the horizontal pathway suggests its unique role in retinal function.

**Figure 5:**
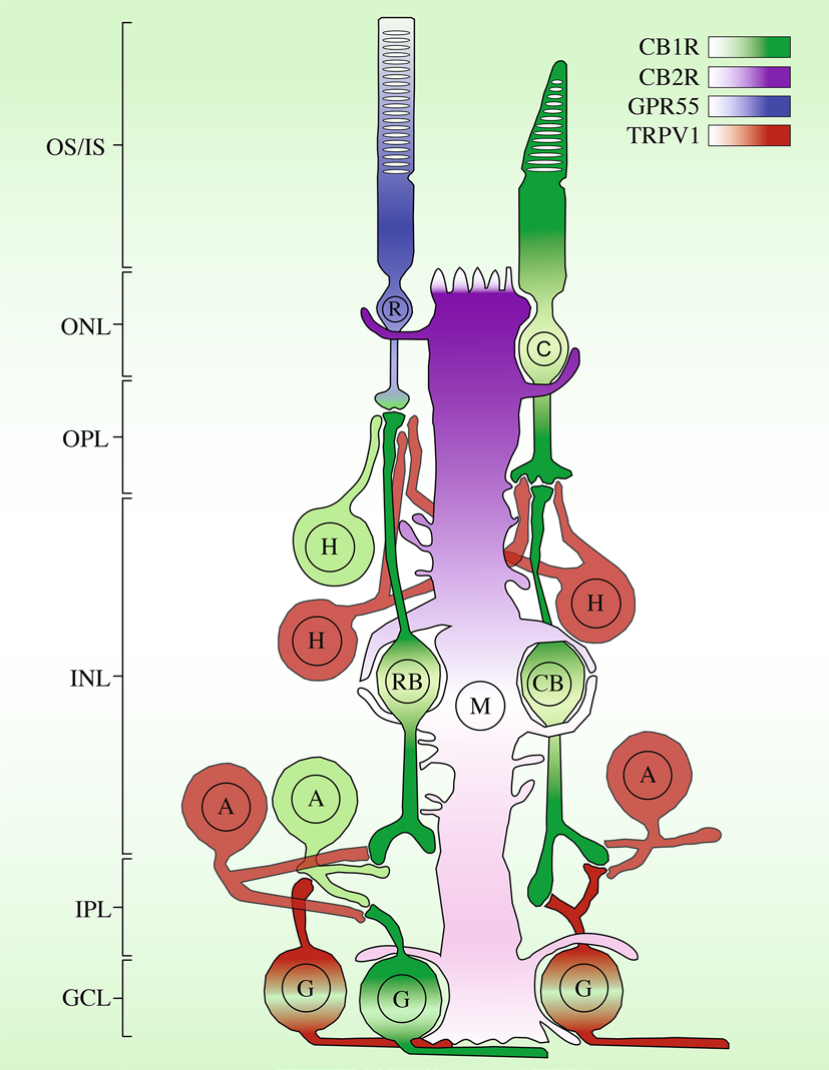
Mapping of the receptors CB1R, CB2R, GPR55, and TRPV1 in the monkey retina. These receptors are differently expressed in the retina of monkeys. These results are compiled from several published articles^26,31,35^. CB1R is represented in green, CB2R in magenta, GPR55 in blue, and TRPV1 in red. OS, photoreceptors outer segments; IS, photoreceptors inner segments; ONL, outer nuclear layer; ONL, outer plexiform layer; INL, inner nuclear layer; IPL, inner plexiform layer; GCL, ganglion cell layer.

We compared TRPV1 labeling with that of the other cannabinoid receptors, CB1R, CB2R, and GPR55 previously described in our laboratory in the vervet monkey retina^26,31,35^. These four receptor types appear to be differentially expressed in the monkey retina. CB1R is observed in the central portion of the retina, from the photoreceptors (cones with heavy staining in the outer segments and pedicles, and rods with little staining confined into their spherules), the horizontal cells, the bipolar cells, the amacrine cells, and the ganglion cells. CB2R is mainly restricted to the retinal glia, the Müller cells. GPR55 is exclusively confined in rods, with a larger signal in its inner segments. As for TRPV1, it is mainly expressed in the horizontal pathway, the horizontal and amacrine cells, but also in ganglion cells. Figure 5 illustrates schematically these data for all retinal cell types.

Importantly, TRPV1 is mainly expressed in the horizontal pathway that contains the inhibitory GABAergic horizontal cells that links photoreceptors and amacrines that connect bipolar cells. The network of TRPV1 channels in horizontal cells, amacrine cells, and RGCs, is likely to be involved in the endocannabinoid and endovanilloid signaling in the retina, which are neuromodulation processes of retinal output^4,21^. The cannabinoid receptors in the vervet monkey retina have been ascribed specific roles in relation to their specific expression. CB1R and CB2R are involved in the modulation of both photopic and scotopic ERG responses^36^ and, GPR55 mediates exclusively scotopic ERG responses^27^. The presence of TRPV1 in inhibitory horizontal pathway might be responsible for modulating information entering the vertical pathway.

### Hypothetical function of TRPV1 in the monkey retina

**Figure 6:**
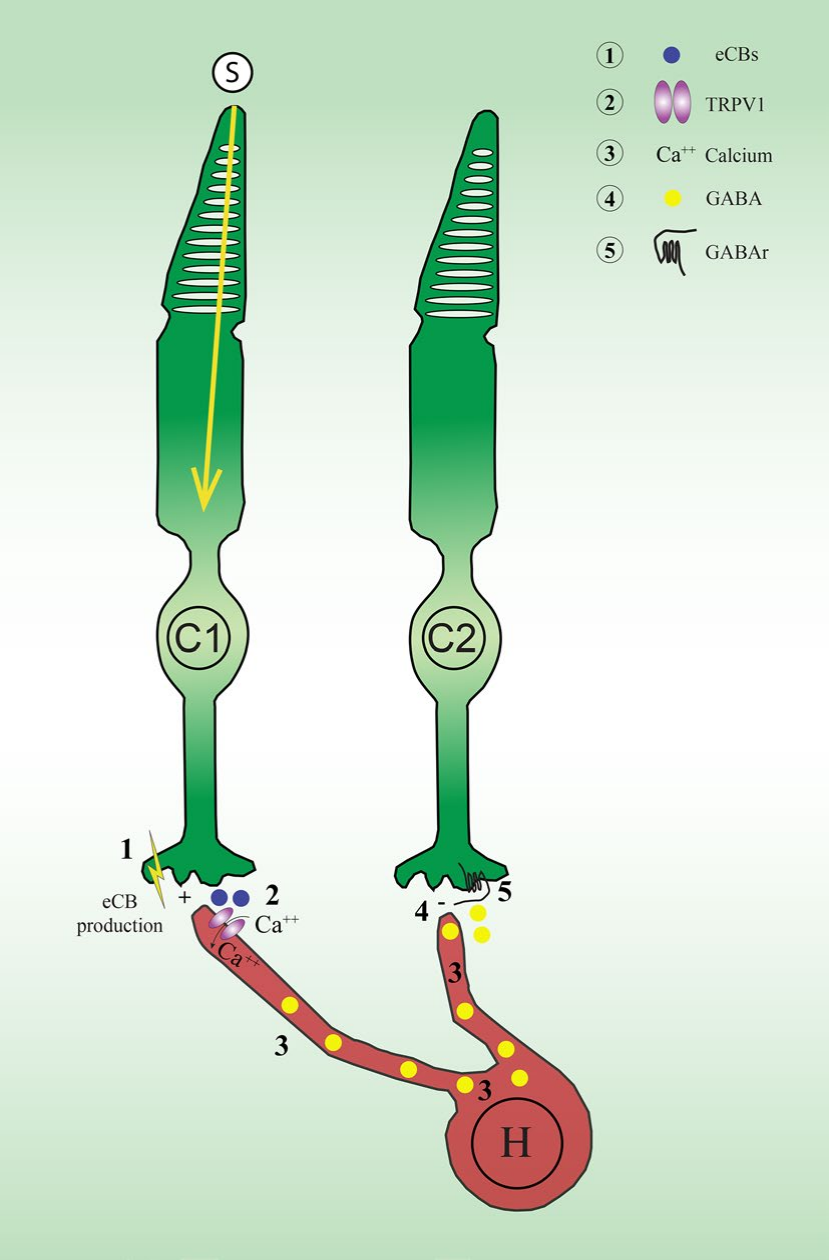
Schematic illustration of the hypothetical mechanism of action of TRPV1 in horizontal cells of the monkey retina following stimulation of a cone. The different steps are highlighted by numbers. (1) When a cone is stimulated (C1), endocannabinoids (eCBs), particularly AEA and 2-AG, are produced in the synaptic cleft. (2) TRPV1 receptors are activated by eCBs in horizontal cells. (3) The calcium influx through the membrane causes depolarization of the horizontal cell. (4) GABA release in the synaptic cleft. (5) GABA binds to GABA receptors on the presynaptic neighboring cone pedicle (5). This mechanism of lateral inhibition could also potentially modulate the information transmitted to the bipolar cells and retinal ganglion cells. *S* spot stimulus, *C1* stimulated cone 1, *C2* adjacent cone 2, *H* horizontal cell, *eCB* endocannabinoid, *GABAr* Gamma-amino-butyric acid receptors, *TRPV1* Transient receptor potential vanilloid type 1.

Horizontal cells (HC) are known to have a modulatory effect on the cone photoreceptors that they connect at the level of their pedicles^37^. It has been suggested that HC can exert their influence through the modulation of the release of the inhibitory neurotransmitter GABA^38^. We propose here another modulatory function of HC, this time, through the activation of the endocannabinoid system present in the photoreceptors (NAPE-PLD for AEA and DAGL for 2-AG). For example, the stimulation of a cone photoreceptor by a discrete small spot evokes the release of endocannabinoids, which in turn activates the TRPV1 receptors on the post-synaptic HC membrane. The Ca^++^ channels are then activated and there is a large calcium influx through the HC membrane. The influx of Ca^++^ depolarizes the HC and stimulates the release of inhibitory GABA in the synaptic cleft. The adjacent cone photoreceptors connected through the HC dendrites are therefore inhibited, which might result in the increased activation of the stimulated cone photoreceptor (Fig. 6). This mechanism involving the endocannabinoid system could be responsible for a first stage lateral inhibition process that influences the cone recipient bipolar cells, and a second stage inhibitory process that involves the amacrine cells (AC). AC connect the bipolar cell dendrites that synapse onto the retinal ganglion cells (RGC) somata. AC modulate the RGC input through a mechanism of action similar to HC since they also contain TRPV1 receptors, as well as GABA and glycine. The presence of the endocannabinoid system in the lateral retina suggests a two-stage inhibitory mechanism that fine-tunes the information processed by retinal ganglion cells.

## Data Availability

The data generated and analyzed during the current study are available from the corresponding author on reasonable request.
